# The dark side of T2: central nervous system lesions with low signal intensity on T2-weighted imaging

**DOI:** 10.1590/0100-3984.2023.0085-en

**Published:** 2024-04-23

**Authors:** Pedro Carpentieri-Primo, Luiza Nahoum, Louise Almeida, Fernando Nacur, Sérgio Ferreira Alves Júnior, Nina Ventura

**Affiliations:** 1 Universidade Federal do Rio de Janeiro (UFRJ), Rio de Janeiro, RJ, Brazil; 2 Grupo Fleury, Rio de Janeiro, RJ, Brazil; 3 Instituto Estadual do Cérebro Paulo Niemeyer, Rio de Janeiro, RJ, Brazil

**Keywords:** Radiology, Central nervous system, Diagnosis, differential, Magnetic resonance imaging, Radiologia, Sistema nervoso central, Diagnóstico diferencial, Ressonância magnética

## Abstract

The majority of central nervous system diseases show high signal intensity on T2-weighted magnetic resonance imaging. Diseases of the central nervous system with low signal intensity are less common, which makes it a finding that helps narrow the differential diagnosis. This was a retrospective analysis of brain and spine magnetic resonance imaging examinations in which that finding was helpful in the diagnostic investigation. We selected the cases of patients examined between 2015 and 2022. All diagnoses were confirmed on the basis of the clinical-radiological correlation or the histopathological findings. We obtained images of 14 patients with the following central nervous system diseases: arteriovenous malformation; cavernous malformation; metastasis from lymphoma; medulloblastoma; embryonal tumor; metastasis from melanoma; Rathke’s cleft cyst; Erdheim-Chester disease; aspergillosis; paracoccidioidomycosis; tuberculosis; syphilis; immunoglobulin G4-related disease; and metastasis from a pulmonary neuroendocrine tumor. We described lesions of different etiologies in which the T2-weighted imaging profile helped narrow the differential diagnosis and facilitated the definitive diagnosis.

## Abstract (pt)

A grande maioria das doenças do sistema nervoso central apresenta alto sinal em ponderações T2 na ressonância magnética. As alterações com baixo sinal são menos comuns, de forma que essa característica permite estreitar o diagnóstico diferencial. Analisamos, retrospectivamente, pacientes com imagens de ressonância magnética de crânio e/ou coluna em que este achado foi útil na investigação diagnóstica. Os pacientes foram selecionados no período entre 2015 e 2022 e todos tiveram seus diagnósticos confirmados por estudo clinicorradiológico ou por estudo histopatológico. Obtivemos imagens de 14 pacientes com as seguintes afecções: malformação arteriovenosa, cavernoma, metástase de linfoma, meduloblastoma, tumor embrionário, metástase de melanoma, cisto da bolsa de Rathke, doença de Erdheim-Chester, aspergilose, paracoccidioidomicose, tuberculose, sífilis, doença relacionada à IgG4 e metástase de tumor neuroendócrino de pulmão. Descrevemos lesões de diversas origens etiológicas que, a partir de suas características nas imagens ponderadas em T2, foi possível reduzir o quadro de diagnósticos diferenciais e chegar mais facilmente à hipótese final.

## INTRODUCTION

The vast majority of central nervous system (CNS) diseases show high signal intensity on T2-weighted magnetic resonance imaging (MRI). Those showing low signal intensity on T2-weighted imaging (T2WI) are less common, which makes that a characteristic that allows the differential diagnosis to be narrowed^(1)^. Low signal intensity on T2WI can be attributed to one of the following^(2)^: rapid blood flow (flow void); high cellularity; high content of protein, melanin, or minerals (calcium, copper, or iron); granulomas; or the presence of certain hemoglobin degradation products. For lesions with high cellularity, the signal intensity is expected to be high on diffusion-weighted imaging (DWI). Lesions for which the low signal intensity on T2WI does not reflect cellularity will typically also show a hypointense signal on DWI^(1)^.

We selected cases from our institution in which the signal intensity on T2WI was useful in the diagnostic investigation. We describe lesions of various etiological origins for which the T2WI characteristics helped reduce the pool of differential diagnoses and facilitated the definitive diagnosis.

## RAPID BLOOD FLOW

**Figure 1:**
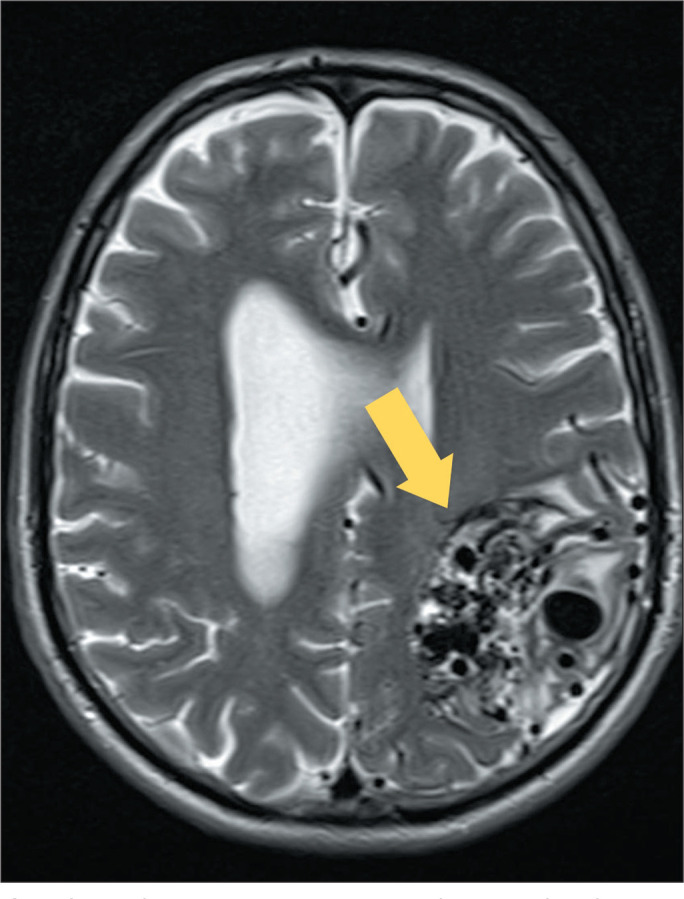
MRI of a patient with arteriovenous malformation. T2WI of the skull, showing a malformation in the left frontoparietal region (arrow), with multiple flow artifacts (flow voids).

Liquids with turbulent flow produce a rapid loss of phase coherence, resulting in low signal intensity on T2WI. That is known as a flow void. The flow void phenomenon allows us to study the blood within vessels and in lesions with high flow, such as aneurysms and arteriovenous malformations^(1)^, as depicted in Figure 1.

## HIGH CELLULARITY

Lesions with high cellularity, such as neoplasms, typically show low signal intensity on T2WI. That finding is related to a disparity in the nucleus/cytoplasm ratio (normally 1:1). Tumors with a high nucleus/cytoplasm ratio, such as small round blue cell tumors, have less extracellular space, which means that the cells are less hydrated, resulting in shorter T2 relaxation times^(2)^.

A high nucleus/cytoplasm ratio also leads to a reduction in the diffusivity of water, which results in high signal intensity on DWI and low signal intensity on apparent diffusion coefficient maps, indicative of restricted diffusion. High tumor cellularity itself also contributes to restricted diffusion on DWI, because the water molecules need to diffuse through a greater quantity of cellular membranes^(3)^.

**Figure 2:**
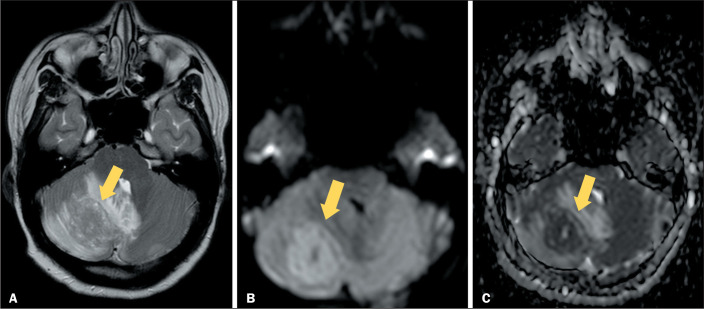
MRI of an 18-year-old patient diagnosed with mediastinal non-Hodgkin lymphoma. A: T2WI of the brain, performed during occipital headache, showing a metastasis in the right cerebellar hemisphere (arrow), with low signal intensity and a significant mass effect on adjacent structures. B,C: DWI and apparent diffusion coefficient map, respectively, showing restricted diffusion and confirming the high cellularity.

A finding of low signal intensity on T2WI is fundamental to the differential diagnosis of CNS tumors, given that glial tumors typically show a hyperintense signal on T2WI, reflecting their high hydration. Posterior fossa tumors, especially those of embryonic origin, such as medulloblastomas and teratoid/rhabdoid tumors, show low signal intensity on T2WI, together with restricted diffusion, reflecting their high cellularity^(4)^. Lymphoproliferative neoplasms, such as lymphoma (Figure 2), can also present in that way, as can post-transplant lymphoproliferative disorder^(5)^.

## MELANIN

**Figure 3:**
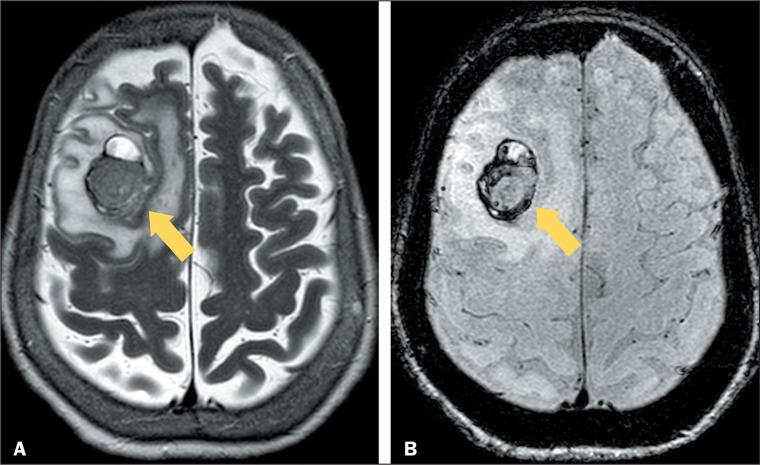
MRI of a patient with metastasis from melanoma. A: T2WI of the skull, showing a metastasis in the right frontal lobe with hypointense content (arrow), peripheral foci corresponding to hemorrhage, whereas the central region corresponds to melanin deposits in the lesion. B: SWI showing magnetic susceptibility artifacts in the periphery due to residual blood products (arrow).

Melanin has paramagnetic properties and shows a hypointense signal on T2WI. Melanoma metastases to the CNS can present in different forms and locations, with a melanotic or amelanotic pattern. When amelanotic, the lesions resemble metastases from other sites, with low signal intensity on T1WI and high signal intensity on T2WI. The melanotic pattern consists of high signal intensity on T1WI and low signal intensity on T2WI. Melanoma metastases are highly vascularized and have a high tendency to bleed^(6)^. Within a tumor, bleeding and melanin content can have similar characteristics. However, bleeding changes the signal over time (Figure 3), depending on the stage of hemoglobin degradation, and presents magnetic susceptibility artifacts on T2*WI^(7)^.

## HEMOGLOBIN DEGRADATION PRODUCTS

A change in signal intensity can also occur because of contamination of the T2*WI signal in substances with magnetic susceptibility, such as blood and calcifications. This can be best seen on T2*WI, on which the changes typically appear greater than they do on T2WI, due to the “blooming” effect. That feature can be used to differentiate between lesions that intrinsically show low signal intensity and those in which the low signal intensity is related to calcifications or bleeding^(2)^.

The signal intensity of intraparenchymal hemorrhage on MRI changes in accordance with the evolution of the hematoma (Table 1). Over time, hemoglobin degrades from oxyhemoglobin to deoxyhemoglobin and then to methemoglobin. Finally, it is broken down into ferritin and hemosiderin.

**Table 1:** Imaging findings in intraparenchymal hemorrhage..

Stage	Time	Hemoglobin degradation product	T2	Diffusion
Hyperacute	< 24 h	Oxyhemoglobin	Hyperintense	Restricted
Acute	1-3 days	Deoxyhemoglobin	Hypointense	Facilitated
Early subacute	> 3 days to 1 week	Intracellular methemoglobin	Hypointense	Facilitated
Late subacute	1 week to months	Extracellular methemoglobin	Hyperintense	Restricted
Chronic	> 14 days^*^	Hemosiderin	Hypointense	Facilitated

* Persisting for months or years.

The magnetic susceptibility effect is responsible for the low signal intensity observed on T2WI when deoxyhemoglobin, methemoglobin, and hemosiderin are present in the intracellular environment, because of hemoconcentration and clot retraction. That effect is intensified when images are obtained by gradient-echo MRI and reduced with spin-echo techniques. Those by-products of hemoglobin lysis are present, respectively, in the acute, early subacute, and chronic phases of bleeding^(7,8)^.

## HIGH PROTEIN CONTENT

Because of their mucoid/protein composition, cysts with high viscosity, such as colloid cysts and Rathke’s cleft cysts, also show low signal intensity on T2WI^(1)^.

### Colloid cysts

Also known as paraphyseal cysts, colloid cysts are unilocular, well-defined, rounded or ovoid in shape, and almost always solitary and small. Although their origin is not perfectly clear, it is assumed that they originate from the embryonic migration of ectopic endodermal tissue. They are typically found in the foramen of Monro, at the top of the third ventricle, and typically cause symptoms only when they obstruct the flow of cerebrospinal fluid in the region, with headache being the most common clinical presentation.

Colloid cysts are composed of a thin fibrous layer of epithelial cells, interspersed with mucin-producing goblet cells, and a gelatinous center. Their presentation on imaging depends on the distribution of their content, varying according to the quantity of cholesterol, mucous material, protein, and water they contain. Their appearance on T2WI reflects the concentration of water: if there is more thick mucoid content than water, the signal will be hypointense in relation to that of the parenchyma^(2)^.

### Rathke’s cleft cysts

**Figure 4:**
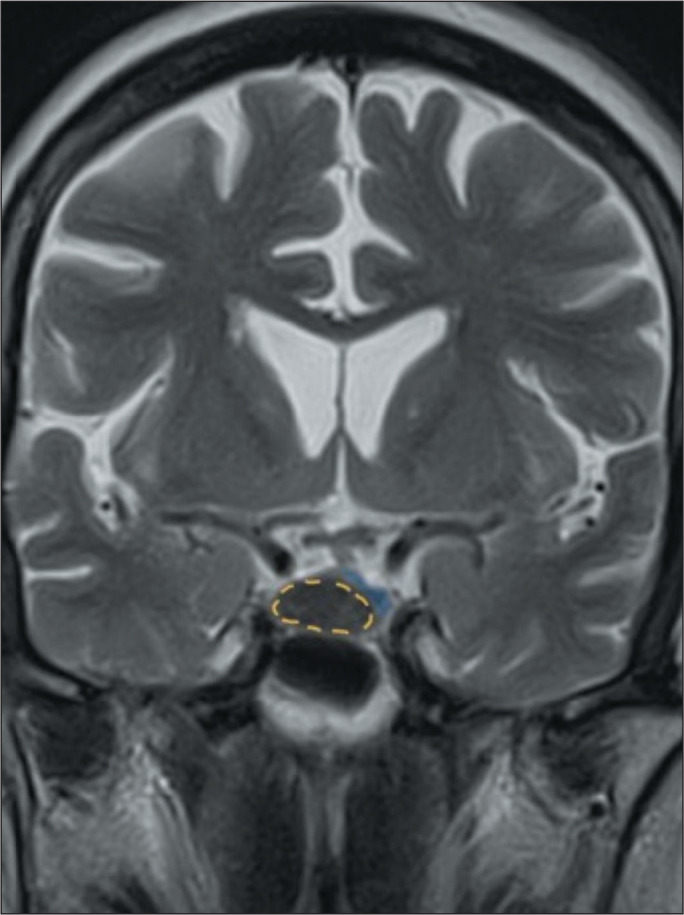
MRI of a Rathke’s cleft cyst in a 63-year-old, asymptomatic patient. Coronal T2WI showing an expansile sellar lesion with a hypointense signal (dotted circle), resulting from its high protein content. The pituitary gland (in blue) is laterally deviated.

Rathke’s cleft cysts are congenital, non-neoplastic cysts arising from the embryological remnants of Rathke’s pouch (Figure 4). They are mainly located in the sellar region but can also be suprasellar. They have a predilection for females, are usually small (less than 3 mm), and do not cause symptoms^(9)^. If they grow too large, they can cause symptoms by compressing adjacent structures such as the optic chiasm and pituitary gland^(9,10)^. Similar to a colloid cyst, a Rathke’s cleft cyst is surrounded by a single layer of ciliated epithelial cells interspersed with goblet cells^(10)^. In some cases, a small intracystic nodule can be seen within it^(9)^. The MRI signal is variable, depending on the content of the cyst. Most Rathke’s cleft cysts show a hyperintense signal on T2WI. However, those containing mucoid material rich in proteins show a homogeneously hypointense signal on T2WI, which is a characteristic aspect of these cysts that is highly suggestive of the diagnosis^(1,9)^.

## NONINFECTIOUS GRANULOMATOUS DISEASES

Granulomatous diseases comprise a family whose common denominator is the histopathological finding of granuloma formation. This family includes infectious diseases such as tuberculosis, and non-infectious diseases such as histiocytosis and sarcoidosis. A granuloma is a focal collection of inflammatory cells, with a predominance of mononuclear cells, generated as a result of the persistence of a non-degradable product and an exacerbated cellular response^(11)^. Granulomas show low signal intensity on T2WI^(11,12)^.

### Sarcoidosis

Sarcoidosis is a systemic disease that, when causing neurological lesions, has a preference for the cranial nerves, as well as for leptomeningeal and dural involvement, in addition to the formation of intraparenchymal granulomas. In sarcoidosis, leptomeningitis has a nonspecific nodular appearance that can also be seen in tuberculosis, lymphoma, and metastases. Dural involvement usually manifests as hypointense dural masses on T2WI^(13)^. Sarcoid granulomas can be found in any part of the parenchyma, although they are most common in the hypothalamic-pituitary region, commonly presenting as plaques or nodular thickening of the optic chiasm and pituitary stalk. Such lesions are usually isointense on T1WI and hypointense on T2WI, with significant contrast enhancement and without restricted diffusion on DWI^(13)^.

### Histiocytoses

Histiocytoses are systemic diseases that affect multiple organs, including the CNS. This group of diseases is characterized by chronic infection with uncontrolled proliferation of macrophages and dendritic cells. Some histiocytoses share imaging findings such as extra-axial involvement of the hypothalamic-pituitary axis and of the calvaria^(12,14)^. The group includes entities such as Langerhans cell histiocytosis, Rosai-Dorfman disease, and Erdheim-Chester disease. All histiocytoses have an affinity for the meninges and often manifest as meningeal thickening with low signal intensity on T2WI. Although the majority show no changes in diffusion, some histiocytoses can mimic neoplasms and show true restricted diffusion^(12,14)^.

*Langerhans cell histiocytosis* - In many cases, Langerhans cell histiocytosis involves the calvarial bones, causing osteolytic lesions with sclerotic margins, and the hypothalamic-pituitary axis, being a classic cause of diabetes insipidus. Less commonly, it can present as granulomatous lesions of the meninges, choroid plexus, pineal gland, or brain parenchyma, simulating a tumor and showing markedly low signal intensity on T2WI^(12)^.

**Figure 5:**
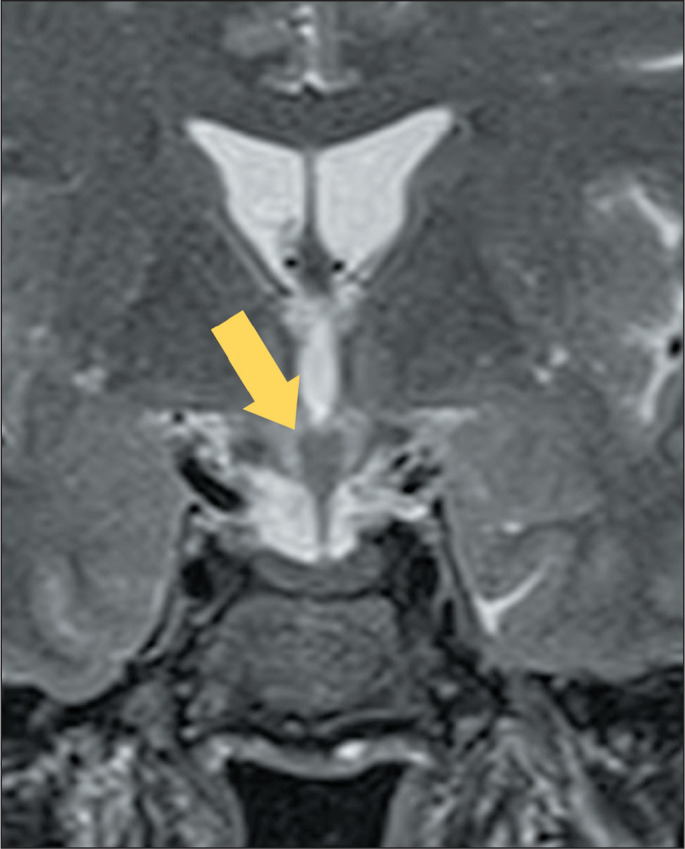
MRI of a patient with Erdheim-Chester disease. Coronal T2WI showing thickening of the infundibular/chiasmatic region of the hypothalamus (arrow), with low signal intensity.

*Erdheim-Chester disease* - Erdheim-Chester disease affects patients ≥ 60 years of age and is more prevalent in men (at a ratio of 3:1). The most common manifestation is involvement of the long bones (seen in approximately 95% of cases), especially in the lower limbs^(12)^. In approximately 50% of cases, it affects the CNS, mainly the hypothalamic-pituitary axis, brain parenchyma, orbits, and meninges (Figure 5). The lesions in the CNS typically show markedly low signal intensity on T2WI and intense homogeneous enhancement by gadolinium. A unique feature of Erdheim-Chester disease that helps differentiate it from other histiocytoses is persistent enhancement in the later stages^(12,14)^. In the orbit, retro-orbital masses can be seen around the optic nerves, as can diffuse infiltration of fat associated with exophthalmos, showing the same hypointense signal on T2WI that is seen in cranial lesions.

*Rosai-Dorfman disease* - Rosai-Dorfman disease is another systemic histiocytosis whose main characteristic is massive lymph node enlargement. Involvement of the CNS is uncommon. The most common pattern on MRI is an isointense or hypointense extra-axial mass on T2WI with homogeneous gadolinium enhancement and low perfusion, the last allowing it to be differentiated from a meningioma, which exhibits high perfusion.

## INFECTIONS

Neuroimaging findings of CNS infections are highly variable, and there is often significant overlap in the appearance of different diseases, which makes it difficult to determine a specific diagnosis^(4)^. Therefore, we highlight a select group of infectious agents that, when producing intracranial disease, tend to generate lesions with low signal intensity on T2WI. However, it should be borne in mind that, in this context, signal intensity is not related to cellularity, as it is in neoplasms, because the capsule of an infectious process typically shows low perfusion and does not show restricted diffusion^(15)^.

### Mycobacteria

**Figure 6:**
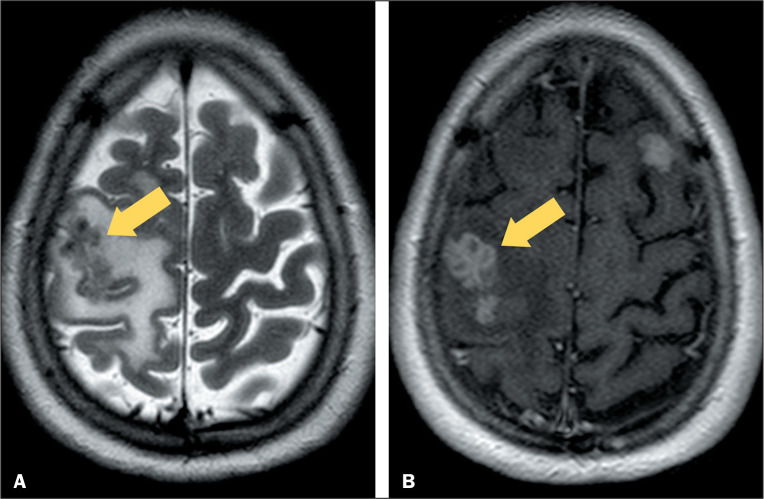
Neurotuberculosis. MRI of the skull, by T2WI (A) and contrast-enhanced T1WI (B), in a 44-year-old patient who tested positive for acid-fast bacillus in cerebrospinal fluid, showing multiple hypointense lesions throughout the leptomeninges bilaterally, with a “bunch-of-grapes” pattern (arrows), together with a hypointense signal on T2WI and ring contrast enhancement, findings that are characteristic of neurotuberculosis.

The main mycobacterial pathogen in Brazil is *Mycobacterium tuberculosis*, which can produce intracranial infection, typically through hematogenous dissemination of pulmonary infection. A tuberculoma, which manifests as a solid area of caseous necrosis and is the most common presentation of tuberculosis in the brain parenchyma, appears hypointense on T2WI^(15,16)^, as can be seen in Figure 6. If the necrosis undergoes liquefaction, the area will show high signal intensity on T2WI^(16)^.

### Spirochetes

The most prevalent spirochetal infection is syphilis, which is caused by sexual or vertical transmission of the spirochete *Treponema pallidum*^(17)^. Radiologically, neurosyphilis can manifest as leptomeningitis, with a thickened, nodular appearance, similar to other granulomatous diseases. It also causes multifocal arteritis, which affects larger vessels more than small vessels and can progress to cerebral infarctions, as well as to nonspecific lesions of the white matter and cerebral gummata^(15,17)^.

**Figure 7:**
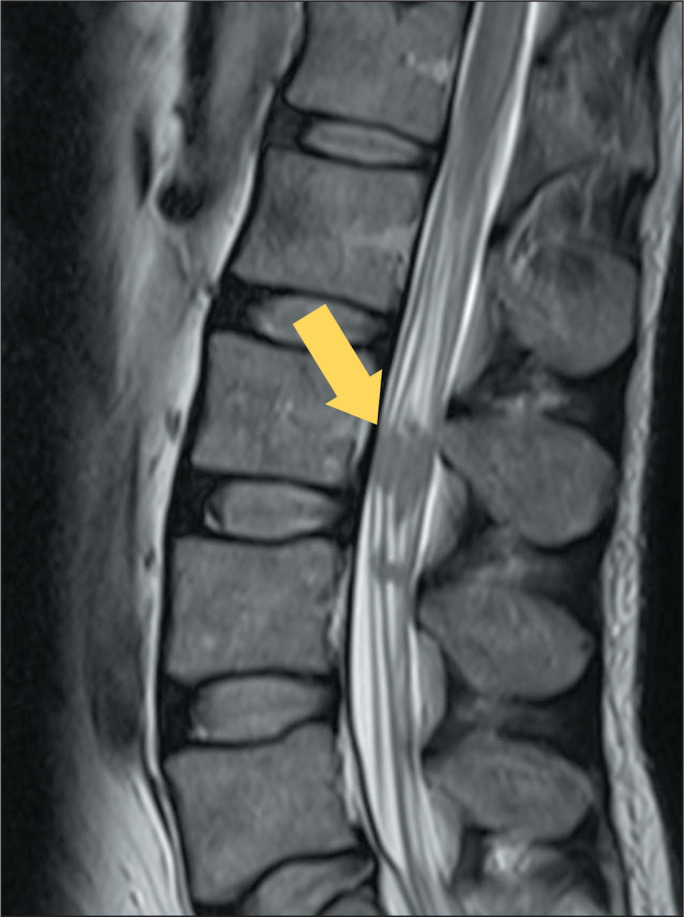
MRI of a patient with neurosyphilis. T2WI of the lumbar spine, showing several hypointense nodular lesions in the roots of the cauda equina (arrow). There was also extensive edema throughout the spine (not shown).

In most cases, syphilitic gummata are peripheral cortical lesions with a dural base that mimic meningiomas. They can also present as bilateral lesions in the mesial temporal lobe, mimicking herpetic encephalitis^(16)^. Syphilitic gummata are typically hypointense on T1WI, with intense contrast enhancement, and are heterogeneously hyperintense on T2WI (Figure 7). However, the neuroimaging findings of syphilis are as variable as are its clinical manifestations, and lesions that are hypointense on T2WI can be confused with those occurring in other neurological disorders^(18)^.

### Protozoa

The protozoan *Toxoplasma gondii* typically produces multiple lesions that are hypointense on T2WI, surrounded by areas of hyperintensity corresponding to intense vasogenic edema. The most specific characteristic of an abscess caused by toxoplasmosis is the presence of the eccentric target sign on contrast-enhanced images, on which ring and nodular enhancement are observed in the periphery of the lesion^(17)^. On fluid-attenuated inversion recovery sequences and T2WI, three zones can be observed^(15)^: a central, hyperintense area (of central necrosis), surrounded by an intermediate, hypointense region (hypervascular zone, in which there are numerous trophozoites, cysts, and inflammatory cells), and delineated by a hyperintense rim (in which there is gliosis, fibrosis, and few trophozoites). This aspect is called the concentric target sign^(19)^.

### Fungi

**Figure 8:**
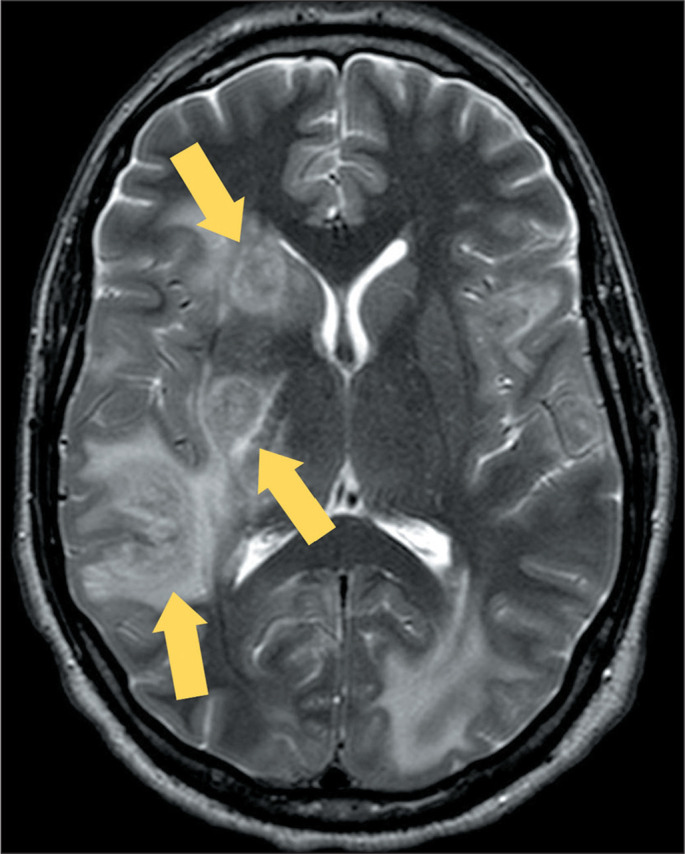
Cerebral aspergillosis. MRI of a 40-year-old patient with acquired immunodeficiency syndrome and a cerebrospinal fluid culture that was positive for *Aspergillus sp*. T2WI showing multiple lesions with low signal intensity, consistent with fungal abscess (arrows).

A variety of fungal pathogens can cause CNS infection. The most common include *Aspergillus fumigatus, Cryptococcus neoformans, Histoplasma capsulatum*, and *Mucor* spp. (which can cause mucormycosis). The extent and severity of infection often depend on the immune status of the patient. These pathogens can cause focal parenchymal lesions, known as fungal granulomas, mycetomas, or “fungus balls” (Figure 8). The lesions are often hypointense on T1WI but can present with shortening of the T1 relaxation time if they are accompanied by subacute hemorrhage, in which case they will also show low signal intensity on T2WI^(16)^. Irregular walls with non-enhancing intracavitary projections are typical findings that correspond to the area of hyphae proliferation^(15,16)^. On T2*WI, there can be focal magnetic susceptibility artifacts caused by hemorrhage and calcification.

**Figure 9:**
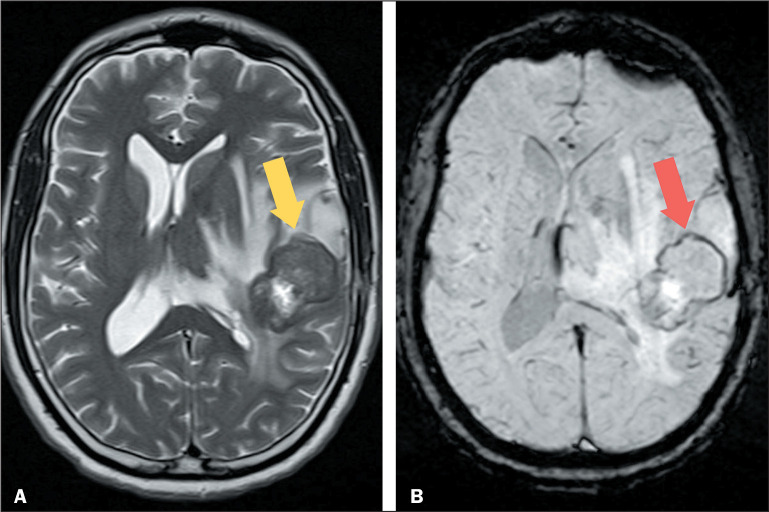
MRI of a patient with biopsy-confirmed cerebral paracoccidioidomycosis. A: T2WI of the skull, showing a large mass in the left frontoparietal region (arrow), with central necrosis and markedly low signal intensity on T2WI. B: Susceptibility weighted image showing a double-ring sign (arrow).

Fungi of the genus *Paracoccidioides* can also affect the CNS (Figure 9). The resulting lesions are typically large (greater than 2.0 cm), with irregular contours and a significant mass effect. On T2WI, such lesions show a hypointense signal, reflecting their granulomatous nature. These lesions can also show the double-ring sign on susceptibility-weighted imaging (SWI). The double-ring sign is described as a hypointense external halo and a hyperintense internal halo, often found in other infectious processes such as bacterial abscesses^(10,16)^.

## MISCELLANEOUS

**Figure 10:**
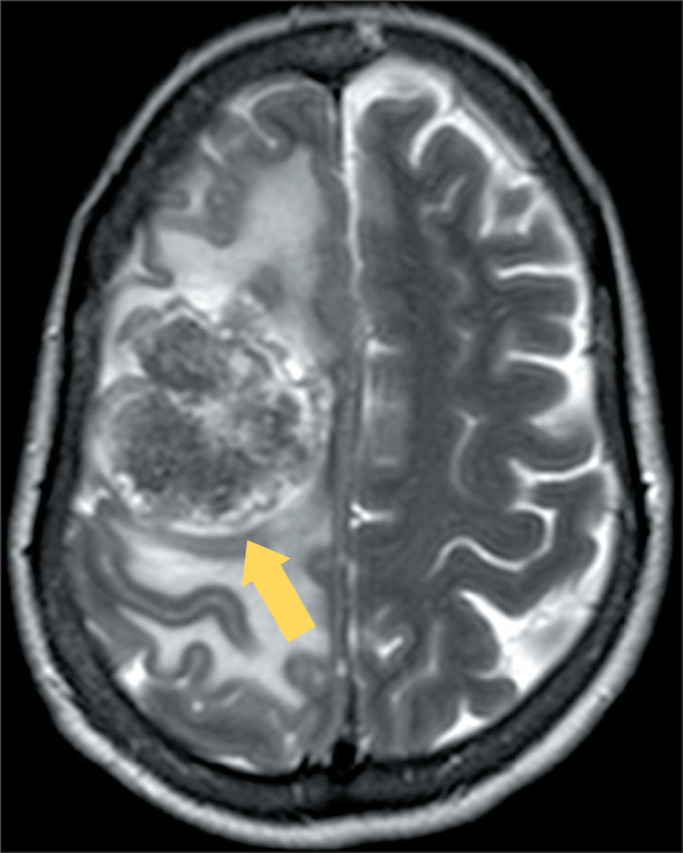
MRI of a patient with metastasis from a pulmonary neuroendocrine tumor. T2WI of the skull, showing a cotton ball-like mass with markedly low signal intensity (arrow).

Some lesions that show a hypointense signal on T2WI still lack a specific anatomical substrate for their characteristic. For example, metastases from non-mucinous adenocarcinomas and neuroendocrine tumors show such signals (Figure 10). It has been suggested that the tumor tissue itself is the source of the signal. However, the physical reason for that finding remains unknown^(20)^.

**Figure 11:**
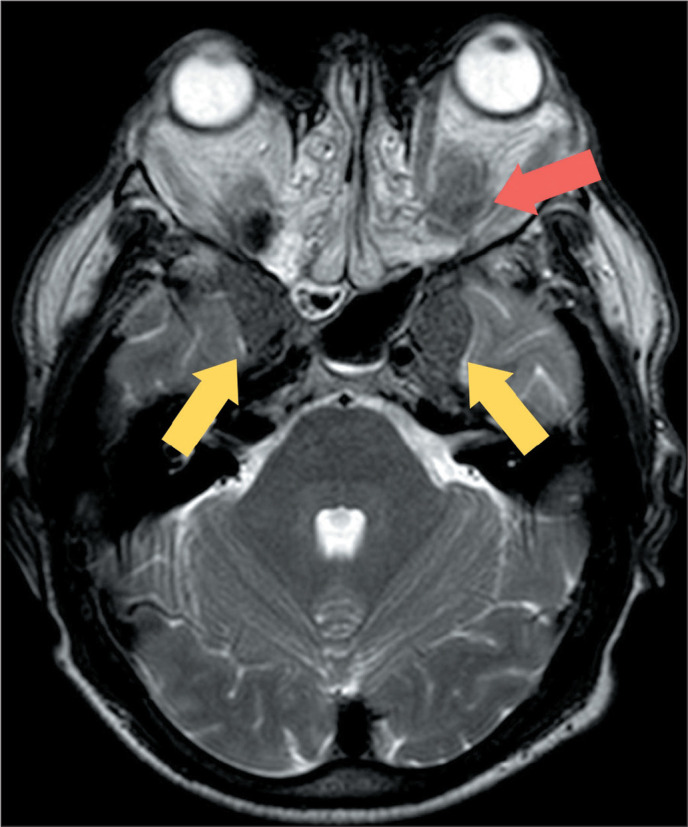
MRI of a patient with IgG4-related disease. T2WI of the skull, showing hypointense tissue infiltrating the optic nerve sheath (orange arrow) and growing through the inferior orbital fissures, round foramen, and oval foramen, subsequently invading the meninges of the middle fossa (yellow arrows), as well as the pterygopalatine recesses and infratemporal fossae.

Immunoglobulin G4-related disease usually presents as an inflammatory mass with a predilection for the lacrimal glands and orbits. The intrinsic signal of the lesion is markedly low on T2WI (Figure 11). It is speculated that the low signal intensity on T2WI is related to the degree of fibrosis caused by a chronic inflammatory process^(21)^.

The presence of a foreign body inside the skull, known as a gossypiboma or textiloma, produces a granulomatous reaction^(22)^. Although the imaging aspect is heterogeneous, it is possible that, depending on the content of the material, there will be hyperintense and hypointense areas on T2WI when there is high fluid and protein content, respectively^(23)^.

The spectrum of diseases related to amyloid-beta protein is wide-ranging, not limited to Alzheimer’s disease alone. The various manifestations include an expansile intra-axial lesion, known as an amyloidoma, that shows contrast enhancement and, in some cases, a hypointense signal on T2WI^(24)^.

## FLOW CHART

**Figure 12:**
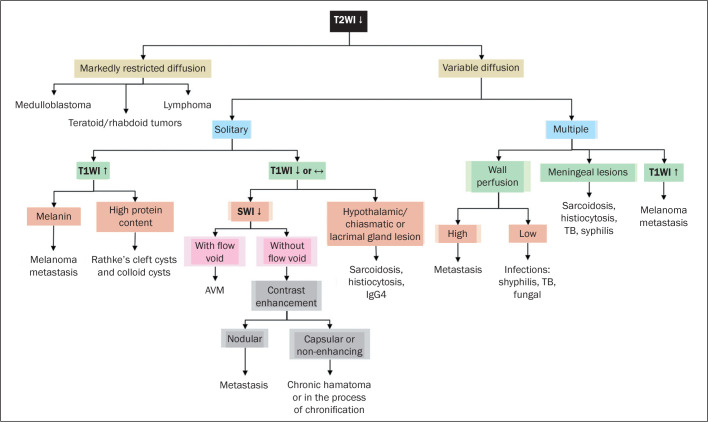
Diagnostic reasoning proposed by the authors and based on the finding of low signal intensity on T2WI.

We propose a flow chart (Figure 12) to be used in order to categorize lesions with low signal intensity on T2WI. According to the findings on additional sequences (especially DWI or SWI sequences) and the anatomical predilection of certain diseases, the diagnosis can be narrowed. The lesions are divided into those with markedly restricted diffusion, for which a hypointense signal on T2WI defines the diagnosis, and those with variable restriction (patterns of restricted or facilitated diffusion), for which the diagnosis does not depend exclusively on low signal intensity on T2WI.

## CONCLUSION

The findings of CNS disease are mostly hyperintense on T2WI. Therefore, when the signal on T2WI is low, it is possible to narrow the differential diagnosis to conditions in which there is rapid blood flow, high cellularity, high protein content, high melanin content, high mineral content, granulomas, or the presence of certain hemoglobin degradation products. Knowledge of this aspect of neuroimaging is an important diagnostic tool, the use of which ultimately results in better patient care.
